# CTSL Promotes Autophagy in Laryngeal Cancer Through the IL6‐JAK‐STAT3 Signalling Pathway

**DOI:** 10.1111/jcmm.70364

**Published:** 2025-02-02

**Authors:** Xueying Wang, Junrong Wang, Lei Wang, Ming Song, Hongxue Meng, Erliang Guo, Susheng Miao

**Affiliations:** ^1^ Department of Otolaryngology Head and Neck Surgery Xiangya Hospital, Central, South University Changsha Hunan China; ^2^ Otolaryngology Major Disease Research Key Laboratory of Hunan Province Changsha Hunan China; ^3^ Clinical Research Center for Laryngopharyngeal and Voice Disorders in Hunan, Province Changsha Hunan China; ^4^ Department of Otorhinolaryngology Head and Neck Surgery Harbin Medical, University Cancer Hospital Harbin Heilongjiang China; ^5^ Department of Pathology Harbin Medical University Cancer Hospital Harbin Heilongjiang China; ^6^ Department of Thoracic Surgery Harbin Medical University Cancer Hospital Harbin Heilongjiang China

**Keywords:** autophagy, CTSL, IL6‐JAK‐STAT3 signalling pathway, laryngeal cancer

## Abstract

Cathepsin L (CTSL) is an important oncogene. However, its mechanism of action in laryngeal cancer is still unclear. This study aims to explore the role of CTSL in laryngeal cancer and its clinical significance. Conducting bioinformatics analysis utilising the Cancer Genome Atlas (TCGA) database and the Gene Expression Omnibus (GEO) database. Performing CCK8 analysis, Western blotting, qRT‐PCR, wound healing assay and transwell invasion assay. Additionally, conducting immunoprecipitation experiments and immunohistochemical staining to investigate the impact of CTSL on cell proliferation, autophagy and related signalling pathways. We observed a significant upregulation of CTSL in laryngeal cancer tissues, and its elevated levels are indicative of poor prognosis in laryngeal cancer patients. The proliferation of laryngeal cancer cells relies on the expression of CTSL, with overexpression of this gene enhancing the proliferative capacity of these cells. Concurrently, CTSL is closely associated with the autophagic levels in laryngeal cancer cells. During the autophagic process mediated by CTSL, the IL6‐JAK‐STAT3 signalling pathway is activated, suggesting that CTSL promotes autophagy through the IL6‐JAK‐STAT3 pathway. Considering the correlation between CTSL and autophagy, we developed and validated a multi‐gene signature. The risk score derived from this signature demonstrates significant potential in predicting various aspects. We found that CTSL upregulates autophagy in laryngeal cancer cells by activating the IL6‐JAK‐STAT3 signalling pathway. By taking into account the autophagy‐regulating role of CTSL, the clinical predictive ability of CTSL in HNSC can be enhanced.

## 1 Introduction

Head and neck cancer is the sixth most common cancer globally, following only lung, stomach, breast, colorectal and cervical cancers. Among them, laryngeal cancer, as one of the most prevalent types of head and neck cancer, continues to see an increasing incidence year by year [1]. The occurrence of laryngeal cancer results from the synergistic effects of various risk factors, including alcohol and tobacco consumption, environmental exposure, improper diet and HPV infection, among others [2]. Laryngeal cancer is prone to lymph node metastasis and frequently leads to respiratory and swallowing function impairment, severely affecting the patients' quality of life and safety. The prognosis is relatively poor, imposing a significant burden and distress on patients and their families [3]. For early‐stage laryngeal cancer, surgery and radiation therapy are the most commonly used treatment methods. Laryngeal cancer is a heterogeneous disease, particularly at the molecular level [4]. In recent years, new drugs and immunotherapy have been continuously developing and may become crucial components of future laryngeal cancer treatments [5]. Despite some progress in research, certain types of laryngeal cancer remain incurable. It is imperative to further explore its biological characteristics and search for early biomarkers and effective therapies.

Laryngeal cancer is a complex disease. The growth and long‐term survival of tumour cells require changes in multiple cellular processes, such as dysregulated angiogenesis, upregulated glycolysis and immune evasion, among others. Autophagy is a catabolic metabolic process in normal cells that provides energy and breaks down large molecular precursors (amino acids, nucleic acids, sugars and fatty acids) [6]. Autophagy plays multiple roles in laryngeal cancer, both supporting the growth and metabolic adaptation of primary tumours and exhibiting opposing effects during the metastatic process, which may affect tumour dormancy, genomic stability and epithelial‐mesenchymal transition. Recent research has also revealed the crucial role of autophagy in the laryngeal cancer microenvironment and immune cells, and some autophagy‐related pathways may have an impact on laryngeal cancer progression. Therefore, the mechanisms behind autophagy and its relationship with laryngeal cancer still require further investigation [7, 8].

Cathepsin L (CTSL) is a lysosomal cysteine protease belonging to the papain‐like family (peptidase C1A), consisting of a heavy and light chain linked by disulphide bonds to form a dimer. It plays a crucial role in intracellular protein degradation and turnover, participating in numerous vital physiological processes, including prohormone activation, antigen presentation and organ development [9]. CTSL is highly expressed in many malignant tumours, including gastrointestinal stromal tumours and metastatic bone tumours, and is considered a potential diagnostic or prognostic marker [10, 11, 12]. Furthermore, studies in the PyMT breast cancer mouse model suggest that CTSL/CTSB strongly influence lung metastasis and exhibit differential effects on the proteome in metastatic lungs. In recent years, there have been reports linking CTSL to drug resistance [13]. Zheng et al. proposed that inhibiting CTSL in drug‐resistant cells contributes to inducing senescence and reversing drug resistance, while CTSL inhibition‐mediated drug target stabilisation may serve as an alternative approach to enhance chemotherapy efficacy [14, 15].

In this study, we investigated whether CTSL is involved in the regulation of autophagy in laryngeal cancer cells and the associated mechanisms. We found that CTSL regulates the IL‐6‐JAK‐STAT3 signalling pathway to enhance autophagy in laryngeal cancer. Furthermore, the integration of CTSL with autophagy‐related genes as a multi‐gene biomarker demonstrates strong predictive power for the prognosis of laryngeal cancer, holding significant clinical utility.

## 2 Materials and Methods

### 2.1 Sample Collection

From 2020 to 2022, human laryngeal cancer tissues and adjacent non‐cancerous tissues were obtained from Harbin Medical University Cancer Hospital. During the surgical procedures, human samples were extracted and preserved in liquid nitrogen for further examination. The collection and utilisation of human samples were approved by the Ethics Committee of Harbin Medical University.

### 2.2 Cell Culture and Transfection

The normal human laryngeal cells (Human Laryngeal mucosal epithelial cells, primary) and laryngeal carcinoma cells (TU212, TU686) were obtained from the iCell Bioscience Inc.(Shanghai, China). Cells were cultured in IMDM (Gibco, USA) or RPMI 1640 (Gibco, USA) medium supplemented with 10% foetal bovine serum (PAN, Germany), penicillin G (100 U/mL, Beyotime, China) and streptomycin (100 μg/mL, Corning, China). Cultures were maintained at 37°C in a humidified incubator with 5% carbon dioxide. For lentivirus transfection, we constructed CTSL overexpression (OE‐CTSL), CTSL knockdown (shCTSL) and empty control (OE‐Control and shNC) using a lentivirus obtained from HANBIO (Shanghai, China). 2 μg/mL puromycin were used to screen out stably constructed laryngeal carcinoma cells. In addition, siRNA from HANBIO (Shanghai, China) was used for transfection of primary normal human laryngeal cells.

### 2.3 Antibodies and Regents

The antibodies used for immunoblot and immunoprecipitation were listed as follows:

anti‐CTSL antibody (27952‐1‐AP, Proteintech, China), anti‐ATG16L antibody (29,445‐1‐AP, Proteintech, China), anti‐Beclin1 antibody(11,306‐1‐AP, Proteintech, China), anti‐LC3‐I/II antibody (ab128025, Abcam), anti‐ATG7 (#8558, CST), anti‐SQSTM1 (#8025, CST), GAPDH (#5174, CST), anti‐IL6 antibody (21865‐1‐AP Proteintech, China), anti‐JAK1 antibody (66466‐1‐Ig, Proteintech, China), anti‐STAT3 antibody (10253‐2‐AP, Proteintech, China), recombinant human IL‐6 protein(HZ‐1019, Proteintech, China).

### 2.4 Proliferation Assay

To conduct the cell proliferation assay, we utilised a Cell Counting Kit‐8(CCK8, MedChemExpress LLC) following the guidelines provided by the manufacturer.

### 2.5 Cell Starvation

Upon reaching 80% confluence, the regular culture medium was substituted with Earle's Balanced Salt Solution (EBSS). The cells were then cultured in EBSS devoid of foetal bovine serum for 8 h to induce autophagy. Subsequently, the cells were harvested in a lysis buffer for Western blotting and qPCR analysis.

### 2.6 Quantitative Real‐Time Polymerase Chain Reaction (qRT‐PCR)

RNA extraction from cells was conducted using TRIzol reagent (Thermo Fisher Scientific, USA), followed by reverse transcription into cDNA using the PrimeScriptTM RT kit (TaKaRa, Japan). For relative quantification through Real‐time PCR, we utilised TB Green PCT Master Mix (TaKaRa, Japan), with GAPDH serving as the reference gene. The qRT‐PCR analysis was performed using the CFX96 real‐time PCR system. The sequences are shown in Table S1.

### 2.7 Western Blot Analysis

Total protein extraction from cells and tissues was carried out using radio‐immunoprecipitation assay (RIPA) buffer (Tymo, Beijing, China). The protein concentration was quantified using a bicinchoninic acid (BCA) kit (Servicebio Technology, Wuhan, China). Subsequently, total protein was separated by electrophoresis and transferred to a membrane. After blocking and three washes with tris‐buffered saline in tween (TBST), the membrane was incubated with primary antibodies (Proteintech, Wuhan, China) overnight at 4°C. Following incubation, the membrane was washed and incubated with secondary antibodies (Proteintech, Wuhan, China). Finally, bands were visualised using an enhanced chemiluminescence (ECL) kit (Servicebio, Wuhan, China).

### 2.8 Wound Healing Assay

Initially, a sterile pipette tip with a volume of 1 mL was used to gently run across the cell surface when the cell density reached 70%. Subsequently, the treated cells were cultured in serum‐free medium. The wound area was observed under an inverted microscope (Olympus, Japan).

### 2.9 Migration Assay

Cells (3 × 10^4^/well) were seeded in serum‐free culture medium in the upper chamber of Transwell inserts (8 μm pore size, Corning, Tewksbury, MA, USA). The lower chamber contained culture medium with 10% FBS as a chemoattractant. After 48 h at 37°C in a 5% CO₂ incubator, migrated cells on the lower side of the insert were fixed with 4% paraformaldehyde, stained with 0.1% crystal violet and imaged under a microscope.

### 2.10 Immunoprecipitation (IP)

293 T cells were harvested, and an immunoprecipitation (IP) lysate containing 50 mM Tris HCl, 1 mM EDTA and 150 mM NaCl was introduced. Subsequently, protein A/G beads were introduced, followed by the addition of antibodies, and the mixture was incubated with gentle shaking at 4°C overnight. The next day, the protein A/G beads were gathered and subjected to three washes with IP buffer. Subsequently, the proteins bound to the beads were eluted by heating in a metal bath at 95°C and subsequently detected using a Western blot assay.

### 2.11 Animal Studies

Male nude mice (4–5 weeks old) were purchased from Vital River Laboratory Animal Technology (Beijing, China). The mice were randomly divided into three groups, each consisting of 5 mice. Subsequently, subcutaneous injections were administered with TU212 cells with downregulated CTSL gene and control group cells, both at a concentration of 5 × 10^6^ cells. The mice were then housed in standard laboratory conditions with adequate food and water supplementation, and tumour size and volume were observed weekly. Prior to sampling, all nude mice were euthanized with 2% pentobarbital sodium (150 mg/kg). After 4 weeks of modelling, all mice were euthanized, and tumour weights were measured. Finally, all tumours were either placed in liquid nitrogen or fixed in 4% paraformaldehyde for further preservation.

### 2.12 Immunohistochemistry (IHC) Staining

After fixation in 4% paraformaldehyde for 24 h, the tumour tissues were embedded in paraffin and cut into 4‐μm‐thick sections. Subsequently, the sections were blocked with 1% bovine serum albumin. Following incubation with primary and secondary antibodies, colorimetric detection was performed using a DAB (diaminobenzidine tetrahydrochloride) reagent kit (CST, USA). The expressions of IL6, JAK and CTSL on the tumour tissue were performed by IHC staining. Tissue sections were incubated with primary antibody at 4°C overnight and then incubated with horseradish peroxidase combined with goat anti‐rabbit antibody (PV‐6001, ZSGB, China) at room temperature for 30 min. Tissue sections were stained using DAB and counterstained with haematoxylin. Finally, the images were captured by Ni‐U upright fluorescence microscope (Nikon, China).

### 2.13 Opal Multiplex Immuno‐Histochemistry

Wax‐embedded paraffin sections from formalin‐fixed specimens were deparaffinised and rehydrated. Subsequently, endogenous horseradish peroxidase (HRP) was blocked using a 3% hydrogen peroxide solution for 30 min. Heat‐induced epitope retrieval was performed in a citrate buffer (95°C, 5 min). Following overnight incubation with the primary antibody at 4°C, fluorescent development was carried out using the Opal 7‐Colour Automation IHC Kit (Akoya Biosciences, Cat # NEL821001KT) following the manufacturer's instructions. The images were captured using the Vectra Polaris Automated Quantitative Pathology Imaging System (Akoya Biosciences, USA) and analysed with inForm Tissue Finder and phenoptr software (Akoya Biosciences, USA).

### 2.14 Data Acquisition

The whole‐transcriptome data and clinical characteristics of Head and Neck Squamous Cell Carcinoma (HNSC) were acquired from The Cancer Genome Atlas (TCGA) project (https:// portal. gdc. cancer. gov/). From the Gene‐Expression Omnibus (GEO) databases, HNSC datasets (GSE58911, GSE58319, GSE84957, GSE41613, and GSE65858) were enrolled. A total of 485 autophagy‐related genes were obtained from the Molecular Signatures Database (MSigDB) autophagy‐related gene sets. Transcriptome sequencing analysis was performed on TU686 CTSL‐KD cell lines and normal TU686 cells, with five replicates for each group (Allwegene, Nanjing).

### 2.15 Construction and Validation of a Prognostic Signature Based on CTSL‐Related Autophagy Genes

We employed univariate COX regression for the preliminary screening of prognostically relevant CTSL‐related autophagy genes. Subsequently, a multivariate COX regression was utilised to further construct an autophagy‐related gene signature. The risk score was calculated as follows: Risk score = ∑ (expression of gene * coef), where coef represents the corresponding coefficients from the multivariate COX regression. Based on the median risk score, we categorised HNSC patients into high‐ and low‐risk groups, followed by various analyses.

### 2.16 Functional Enrichment Analyses and Genome Enrichment Analysis (GSEA)

We utilised the ‘ClusterProfiler’ R package for performing Gene Ontology (GO) analysis, which covers molecular function (MF), biological process (BP) and cellular components (CC). Additionally, we conducted a gene set enrichment analysis (GSEA) to investigate potential molecular mechanisms distinguishing the high‐risk and low‐risk groups.

### 2.17 Statistical Analysis

Statistical analysis was conducted using SPSS version 25 (SPSS Inc., Chicago, IL, USA). The experimental results obtained in both in vitro and in vivo settings were expressed as the mean ± standard deviation (SD). To assess differences between two groups, the Student's *t*‐test was employed. When comparing multiple groups, either one‐way or two‐way analysis of variance (ANOVA) was performed, followed by post hoc Student‐Neuman‐Keuls (SNK) tests to establish means separation. Statistical significance was considered achieved at a significance level of *p* < 0.05. Bioinformatic analysis was carried out using R software (version 4.1.0; https://www.r‐project.org/). *p*‐value < 0.05 were considered statistically significant. The symbol * indicates *p* < 0.05, the symbol ** represents *p* < 0.01, the symbol *** represents *p* < 0.001 and the symbol **** indicates *p* < 0.0001.

## 3 Results

CTSL is significantly upregulated in laryngeal cancer tissues, its elevated levels are indicative of poor prognosis in patients with laryngeal cancer.

**FIGURE 1:**
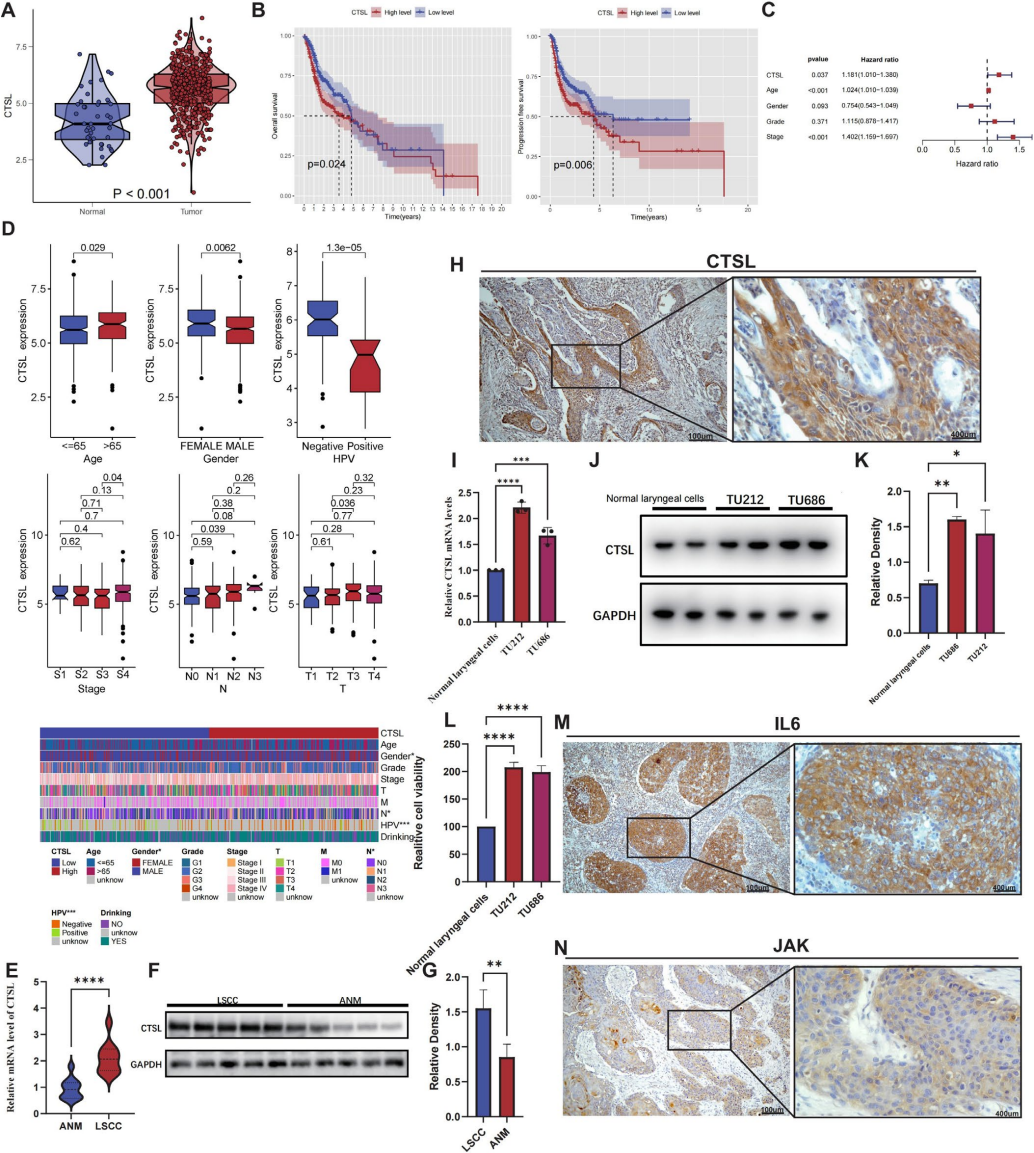
CTSL is significantly upregulated in laryngeal cancer tissues, its elevated levels are indicative of poor prognosis in patients with laryngeal cancer. (A) There is a significant difference in the expression of CTSL between cancer and adjacent tissues in HNSCC patients in the TCGA database. The expression of CTSL in cancer tissues is markedly higher than in adjacent tissues. (B) Kaplan–Meier survival analysis indicates that patients with high CTSL expression have shorter overall survival and disease‐free survival. (C) Univariate Cox analysis reveals that high levels of CTSL expression are an adverse factor influencing patient survival. (D) Among clinically diverse patients, CTSL expression varies. Patients who are older, female, HPV‐negative, at a higher stage, and with poorer TN staging generally exhibit higher CTSL expression. (E) Relative mRNA levels of CTSL in tumour and adjacent tissues of clinical samples, CTSL is highly expressed in tumour tissues. (F) In clinical samples, Western blotting was used to detect the protein expression levels of CTSL in five corresponding tumour tissues and five adjacent normal tissues, with GAPDH used as the internal reference. (G) WB Quantification. (H) CTSL in the immunohistochemical results of laryngeal cancer tissue. (I) Relative mRNA levels of CTSL in normal laryngeal cells and laryngeal cancer cell lines. (J) Western blotting analysis of CTSL protein expression levels in normal laryngeal cells and laryngeal cancer cell lines. GAPDH was used as an internal reference. (K) WB Quantification. (L) CCK8 experiment analysis was conducted in normal laryngeal cells and laryngeal cancer cell lines. (M, N) IL6 and JAK in the immunohistochemical results of laryngeal cancer tissue.

We obtained data from the TCGA and GEO database for HNSCC patients, and the results indicated that the expression level of CTSL in HNSCC tissues was higher than that in normal tissues (Figure 1A, Figure S1A–C). To determine the impact of CTSL expression on the prognosis of HNSC patients, we conducted the analysis revealing a significant correlation between higher CTSL expression and lower overall survival and disease‐free survival rates (Figure 1B, Figure S1D,E). Univariate Cox regression analysis further demonstrated that CTSL is a significant risk factor influencing the survival of HNSCC patients (Figure 1C, Figure S1F,G). Subsequently, we explored the correlation between CTSL expression and clinical parameters. Patients with different clinical presentations exhibited variations in CTSL expression (Figure 1D). The ESTIMATE and CIBERSORT were employed to calculate the close correlation between CTSL expression and immunity. Across multiple GEO datasets, the expression of CTSL consistently demonstrated a close association with the immunity (Figure S1H–O). We measured the expression levels of CTSL in specimens from human laryngeal cancer and adjacent normal tissues. Both mRNA and protein expression levels of CTSL in laryngeal cancer specimens were significantly higher than those in adjacent normal tissues (Figure 1E–G). Immunohistochemical staining of laryngeal cancer tissue also suggests that the area of CTSL‐positive cells is high in laryngeal cancer tissue (Figure 1H).

Furthermore, at the cellular level, the protein and mRNA expression levels of CTSL showed higher expression in laryngeal cancer cells compared to normal laryngeal cells (Figure 1I–K). CCK‐8 assays confirmed that TU212 and TU686 had stronger proliferative capabilities compared to normal laryngeal cells (Figure 1L). In addition, the area of IL6‐positive cells and JAK‐high cells is also high in laryngeal cancer tissue. (Figure 1M,N).

### 3.1 The Proliferation of Laryngeal Cancer Cells Is Dependent on the Expression of CTSL

**FIGURE 2:**
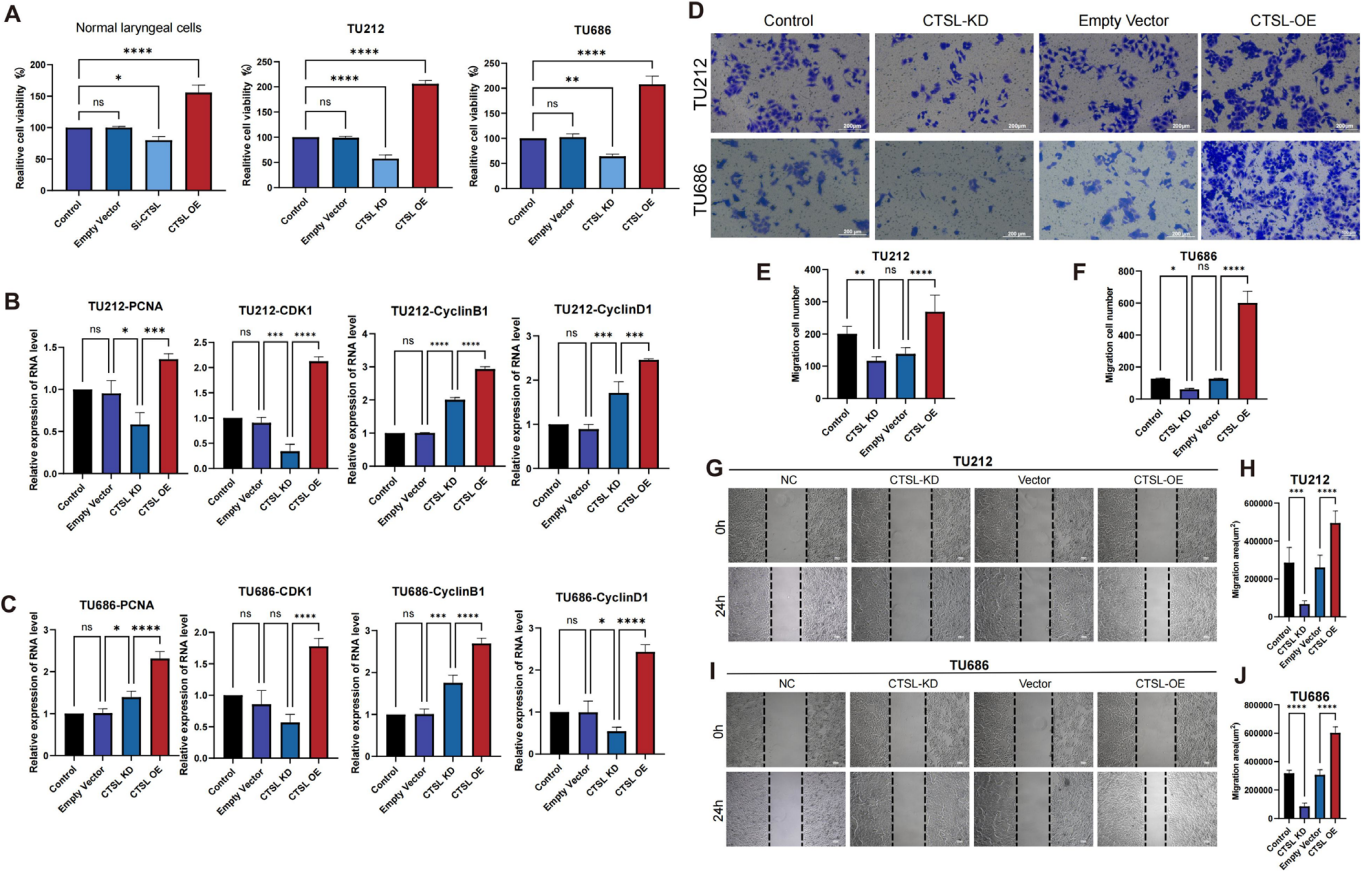
The proliferation of laryngeal cancer cells is dependent on the expression of CTSL. (A) In laryngeal cells and laryngeal cancer cells TU212 and TU686, high expression of CTSL enhances cell proliferation capability, while low expression of CTSL inhibits cell proliferation. (B, C) MRNA levels of cell proliferation‐related markers in TU212 and TU686 cells with control, empty vector, CTSL knockdown (KD) and CTSL overexpression (OE) cell lines. (D–F) Based on transwell experiments, laryngeal cancer cells with CTSL knockdown show reduced invasive ability, while CTSL overexpression enhances the invasive capability of laryngeal cancer cells. (G–J) Representative images of laryngeal cancer cell migration based on scratch assay experiments and their quantification.

After constructing stable cell lines with CTSL knockdown and overexpression using recombinant lentivirus, CCK‐8 assays were employed to validate the impact of CTSL expression on the proliferation of laryngeal cancer cells. Whether in normal laryngeal cells or TU212 and TU686 cells, elevated CTSL expression enhanced cell proliferation, while cells with low CTSL expression showed inhibited proliferation capabilities (Figure 2A). Subsequently, we confirmed the mRNA levels of proliferation‐related markers PCNA, CDK1, CyclinB1 and CyclinD1 in different cell lines. The cell lines overexpressing CTSL exhibited significantly increased mRNA levels of these proliferation‐related markers, further confirming the close association between laryngeal cancer cell proliferation and CTSL expression (Figure 2B,C). Transwell assays demonstrated that CTSL knockdown weakened the invasive capacity of laryngeal cancer cells, while CTSL overexpression enhanced their invasive ability (Figure 2D–F). Scratch assays indicated that CTSL knockdown reduced the migration ability of laryngeal cancer cells, whereas CTSL overexpression increased their migration capability (Figure 2G–J).

### 3.2 Laryngeal Cancer Is Associated With Autophagy

**FIGURE 3:**
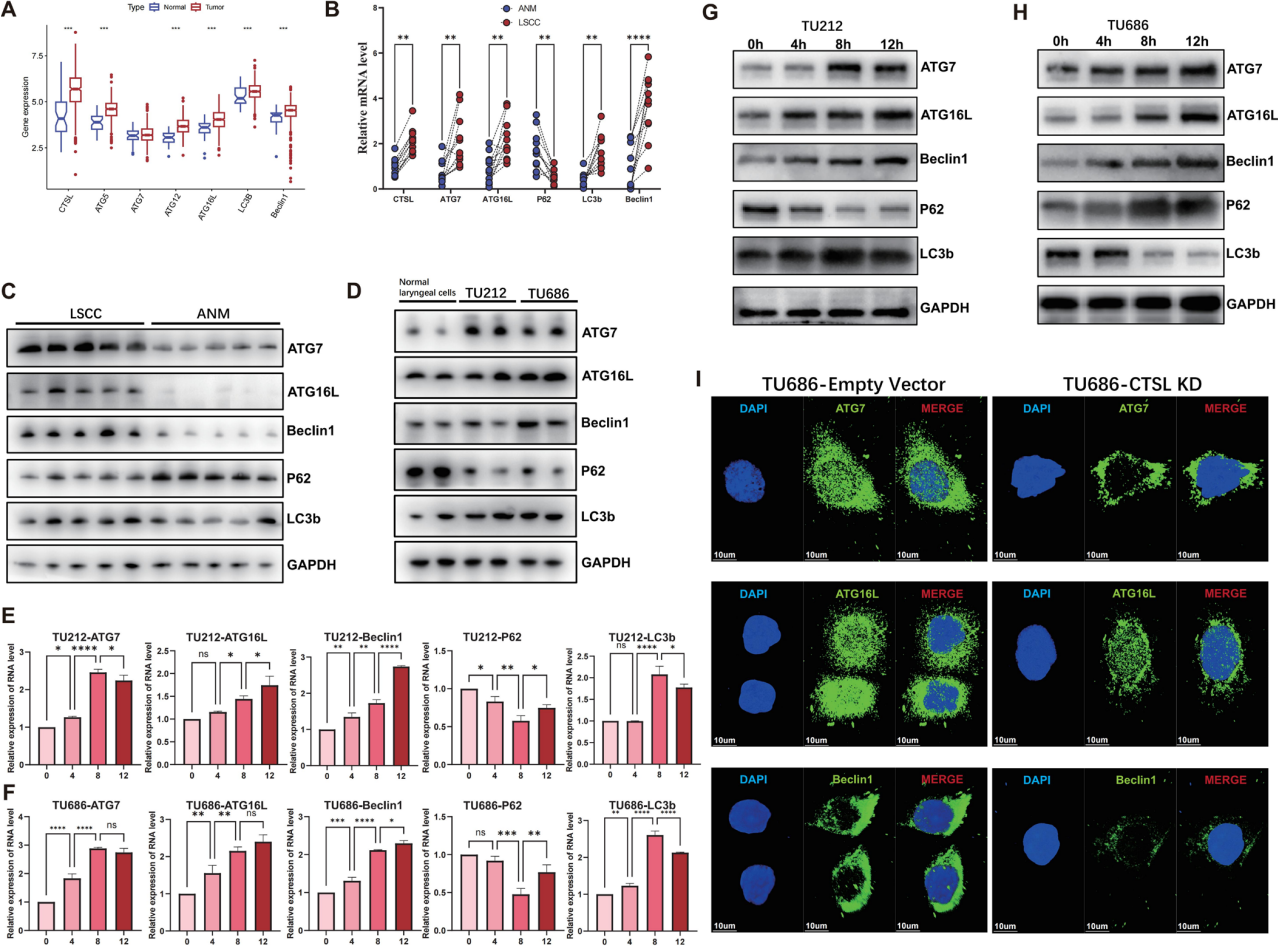
Laryngeal cancer is associated with autophagy. (A) There is a significant difference in the expression of autophagy‐related markers between cancer and adjacent tissues in HNSCC patients in the TCGA database. (B) Paired qPCR monitoring of laryngeal cancer tissues. The mRNA expression of autophagy‐related markers such as ATG7, ATG16L, LC3b and Beclin1 increases in cancer tissues, while p62 decreases. (C) Western blot monitoring of laryngeal cancer and adjacent tissues. The first five lanes represent cancer tissues, and the last five lanes represent cancer adjacent tissues. The protein expression of autophagy‐related markers, including ATG7, ATG16L, LC3b and Beclin1, increases in cancer tissues, while p62 decreases. (D) Western blot monitoring of normal laryngeal cells and laryngeal cancer cells TU212 and TU686. The first two lanes represent normal laryngeal cells, and the last four lanes represent TU212 and TU686. The protein expression of autophagy‐related markers (including ATG7, ATG16L, LC3b and Beclin1) increased in laryngeal cancer cells, while p62 decreased. (E, F) Detection of autophagy in cells under starvation at different time points. Cells were starved in EBSS for 4, 8 and 12 h to induce autophagy before analysis. Cell lysates were collected, and qPCR was used to detect autophagy markers. (G, H) WB was used to detect autophagy markers. (I) Immunofluorescence staining was performed targeting autophagy‐related markers ATG7, ATG16L and Beclin1 in laryngeal cancer cells TU686‐Empty Vector and TU686‐CTSL KD.

Under normal physiological conditions, cells primarily maintain homeostasis through basal levels of autophagy. However, when cells encounter various stressors such as starvation [16], hypoxia [17] or cytotoxic therapy, they activate autophagy as a protective response. The assessment of autophagic activity can be conducted by observing changes in the expression levels of key autophagy‐related proteins. Commonly used autophagy‐related markers include ATG5, ATG7, ATG12, ATG16L, LC3b, P62, Beclin1, etc. [18]. These markers play crucial roles in the autophagic process, with ATG5 and ATG7 particularly playing key roles in autophagosome formation. The expression level of LC3b is usually proportional to the formation of autophagosomes, while p62 is inversely related to autophagosome formation. Analysis of the TCGA database indicates that autophagy‐related markers in laryngeal cancer tissues, such as ATG5, ATG12, ATG16L, LC3b, Beclin1, mostly exhibit an upregulation trend (Figure 3A). Paired tissue qPCR results confirm this observation, showing significantly higher mRNA levels of autophagy‐related markers in laryngeal cancer specimens compared to adjacent normal tissues, with p62 levels lower than those in normal tissues (Figure 3B). Western Blot experiments demonstrate an increase in protein expression levels of autophagy‐related markers in laryngeal cancer tissues (Figure 3C). Similar observations are made in cell lines: compared to normal laryngeal cells, autophagy‐related markers such as ATG7, ATG16L, Beclin1 and LC3b protein expression levels are elevated in laryngeal cancer cells, while p62 expression levels decrease (Figure 3D). Starved laryngeal cancer cells show a significant mRNA increase in autophagy‐related markers such as ATG7, ATG16L, Beclin1 and LC3b at different time points, with a corresponding decrease in p62 levels, indicating the occurrence of autophagy (Figure 3E,F). The protein expression of autophagy‐related markers in the WB experiment results also confirmed this finding (Figure 3G,H). Using immunofluorescence staining to target autophagy‐related markers ATG7, ATG16L and Beclin1 in TU686‐Empty Vector and TU686‐CTSL KD cells, confirming the presence of autophagy in laryngeal cancer cells, Knocking down CTSL can reduce the expression of autophagy‐related markers in laryngeal cancer cells. (Figure 3G).

### 3.3 CTSL Is Closely Associated With the Autophagy Levels of Laryngeal Cancer Cells

**FIGURE 4:**
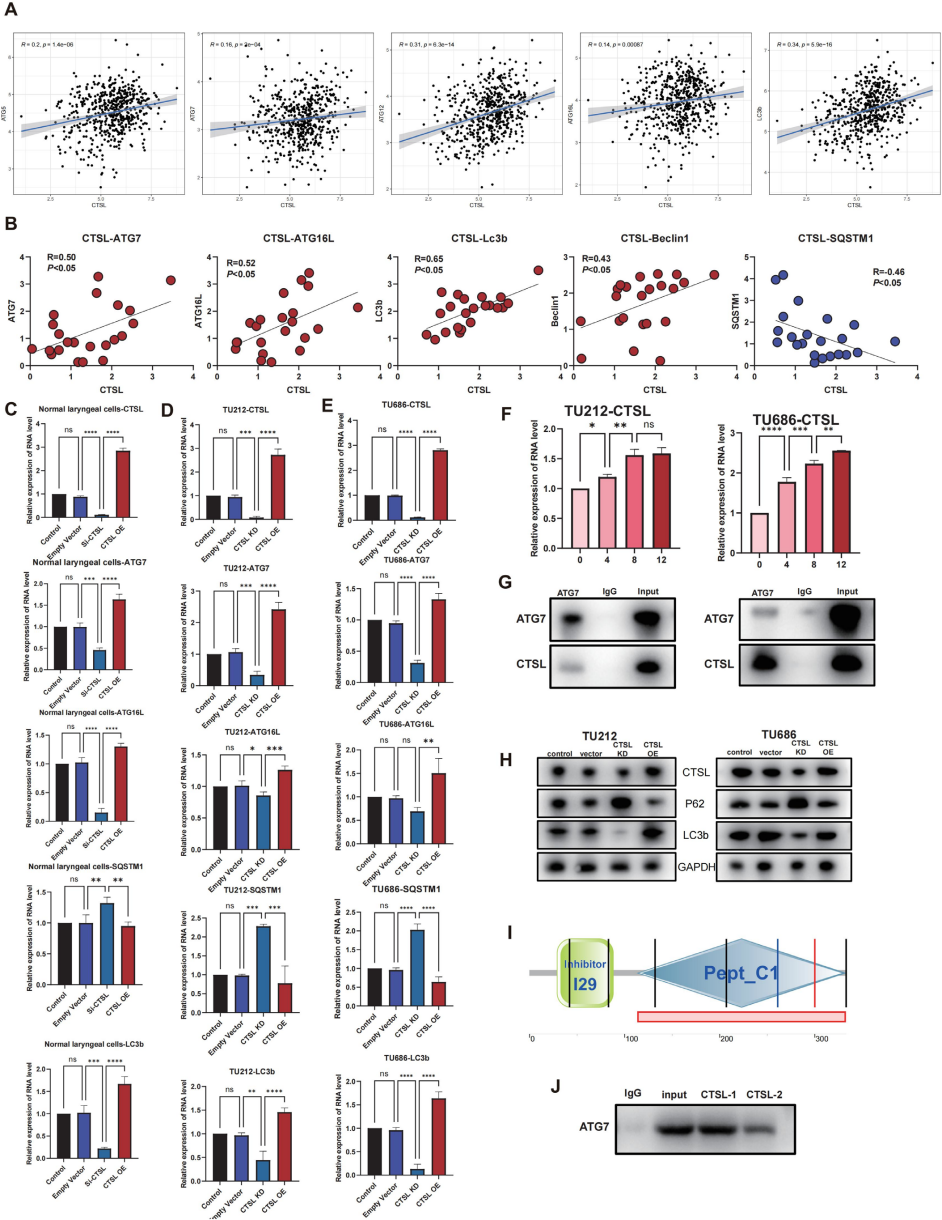
CTSL is closely associated with the autophagy levels of laryngeal cancer cells. (A) Data analysis of TCGA‐HNSC patients reveals a positive correlation between CTSL and autophagy‐related markers ATG5, ATG7, ATG12, ATG16L and LC3b expression. (B) QPCR results show a positive correlation between CTSL mRNA expression and ATG7, ATG16L, LC3b and Beclin1, and a negative correlation with p62. (C–E) Knockdown of CTSL in normal laryngeal cells and laryngeal cancer cells leads to a decrease in mRNA expression of autophagy‐related markers ATG7, ATG16L and LC3b, and an increase in p62. Overexpression of CTSL results in an increase in mRNA expression of ATG7, ATG16L and LC3b, and a decrease in p62. (F) CTSL expression in laryngeal cancer cells undergoing autophagy after starvation shows an increasing trend at different time points. (G) Co‐immunoprecipitation experiments verify the interaction between CTSL and ATG7. (H) In CTSL knockdown laryngeal cancer cell lines, there is an increase in p62 protein expression, while in CTSL overexpressing cell lines, there is a decrease in p62 protein expression. (I) The SMART database revealed two truncated forms of CTSL. (J) CO‐IP experiments with ATG7 shown that the interaction between ATG7 and CTSL predominantly occurs with CTSL‐1.

The data analysis of the TCGA‐HNSC reveals a positive correlation between the expression of CTSL and autophagy‐related markers such as ATG5, ATG7, ATG12, ATG16L and LC3b, suggesting a connection between CTSL and autophagy in laryngeal cancer (Figure 4A). This observation is further confirmed by qPCR, showing a positive correlation between CTSL mRNA expression levels and several autophagy‐related markers, along with a negative correlation with p62 expression (Figure 4B). In Figure 4C, it is demonstrated that knocking down CTSL in normal laryngeal cells leads to a significant decrease in the mRNA expression of multiple autophagy‐related markers, including ATG7, ATG16L and LC3b, while p62 expression significantly increases. Conversely, overexpressing CTSL results in an upregulation of mRNA expression for ATG7, ATG16L and LC3b, accompanied by a decrease in p62 expression. Similar observations are made in laryngeal cancer cell lines TU212 and TU686 (Figure 4D,E). In starving laryngeal cancer cells undergoing autophagy, CTSL expression shows an increasing trend at different time points, providing further evidence of the close association between CTSL and the autophagic levels in laryngeal cancer cells (Figure 4F). Subsequently, co‐immunoprecipitation experiments in 293 T cells and tissues validate the interaction between ATG7 and CTSL (Figure 4G). Western blot experiments also reveal differences in the protein expression of the autophagy‐related marker p62, LC3b in laryngeal cancer cell lines with CTSL knockdown and overexpression (Figure 4H). The SMART database revealed two truncated forms of CTSL (Figure 4I). To further investigate the functions of CTSL and its truncated forms (CTSL‐1, CTSL‐2), we performed overexpression of the truncate using a CTSL truncate overexpression plasmid carrying a GFP tag in 293 t cells. The overexpression of truncated forms was validated through CO‐IP experiments with ATG7. We found that the interaction between ATG7 and CTSL predominantly occurs with truncated form 1 (Figure 4J).

### 3.4 CTSL Promotes Autophagy in Laryngeal Cancer Through the IL6‐JAK‐STAT3 Signalling Pathway

**FIGURE 5:**
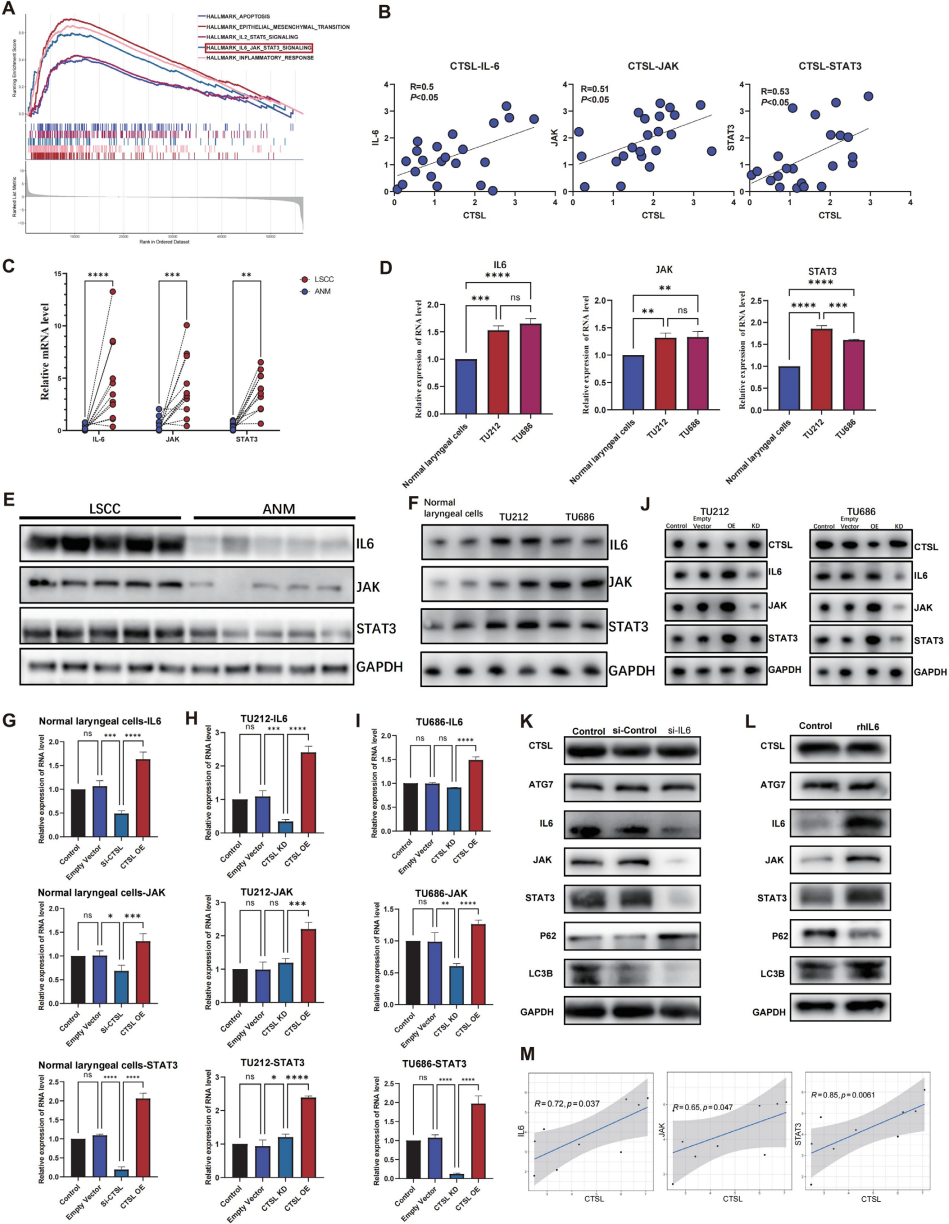
CTSL promotes autophagy in laryngeal cancer through the IL6‐JAK‐STAT3 signalling pathway. (A) Gene Set Enrichment Analysis (GSEA) reveals a significant association between CTSL and the IL6‐JAK‐STAT3 signalling pathway. (B) QPCR results show a positive correlation between CTSL mRNA expression and IL6, JAK and STAT3. (C) Paired qPCR results of cancer tissues and adjacent tissues indicate an elevation in mRNA expression of IL6, JAK and STAT3 in cancer tissues, suggesting increased activity of the IL6‐JAK‐STAT3 pathway. (D) QPCR results in normal laryngeal cells and laryngeal cancer cells show higher activity of the IL6‐JAK‐STAT3 pathway in laryngeal cancer cells (TU212 and TU686) compared to normal laryngeal cells. (E, F) Protein levels of the IL6‐JAK‐STAT3 pathway significantly increase in laryngeal cancer tissues and cells. (G–J) In normal laryngeal cells and laryngeal cancer cells, high expression of CTSL increases the levels of the IL6‐JAK‐STAT3 pathway, while low expression decreases its activity. (K, L) Knockdown of IL6 in CTSL overexpression stable cell lines results in decreased protein expression in the IL6‐JAK‐STAT3 signalling pathway. The protein expression of autophagy‐related marker LC3b decreases, and p62 expression increases. Introducing recombinant IL6 protein into CTSL overexpression stable cell lines increases protein expression in the IL6‐JAK‐STAT3 signalling pathway, while simultaneously increasing LC3b protein expression and decreasing p62 expression. (M) Correlation analysis of CTSL with IL6, JAK and STAT3 in transcriptome sequencing.

In order to explore the downstream signalling pathways of CTSL, we conducted GSEA using HNSC data from the TCGA database. The GSEA results revealed a significant association between CTSL and the IL6‐JAK‐STAT3 signalling pathway (Figure 5A). Our qPCR results confirmed a positive correlation between CTSL mRNA expression and the expression of IL6, JAK and STAT3 (Figure 5B). Paired qPCR results comparing cancer and adjacent tissues indicated elevated mRNA expression of IL6, JAK and STAT3 in cancer tissues, along with an increase in the activity of the IL6‐JAK‐STAT3 pathway, compared to adjacent tissues (Figure 5C). Subsequently, we observed higher activity of the IL6‐JAK‐STAT3 pathway in TU212 and TU686 laryngeal cancer cells compared to normal laryngeal cells (Figure 5D). Western Blot experiments showed a noticeable increase in the protein levels of the IL6‐JAK‐STAT3 pathway in laryngeal cancer tissues and cells (Figure 5E,F). Following the manipulation of CTSL expression in normal laryngeal cells and laryngeal cancer cells, we monitored the expression of mRNA and proteins in the IL6‐JAK‐STAT3 pathway. High expression of CTSL in normal laryngeal cells increased the levels of the IL6‐JAK‐STAT3 pathway, while low expression decreased its activity. Similarly, in laryngeal cancer cells TU212 and TU686, stable transfectants with high CTSL expression exhibited an increase in IL6‐JAK‐STAT3 pathway activity, while low expression stable transfectants showed a decrease (Figure 5G–J). To further investigate whether CTSL promotes autophagy in laryngeal cancer through the IL6‐JAK‐STAT3 signalling pathway, we knocked down IL6 in CTSL overexpression stable transfectants. We observed that as IL6 was knocked down, the expression of proteins in the IL6‐JAK‐STAT3 signalling pathway decreased, along with a reduction in the protein expression of the autophagy‐related marker LC3b and an increase in p62 expression. Additionally, the introduction of recombinant IL6 protein into CTSL overexpression stable transfectants resulted in an increase in the expression of proteins in the IL6‐JAK‐STAT3 signalling pathway, accompanied by an elevation in the protein expression of LC3b and a decrease in p62 expression (Figure 5K,L). These results suggest that CTSL may promote autophagy in laryngeal cancer through the IL6‐JAK‐STAT3 signalling pathway. Additionally, transcriptome sequencing analysis of the TU686 CTSL‐KD cell line and normal TU686 cells showed that IL6, JAK and STAT3 levels increased with the rise of CTSL, further confirming the above results.(Figure 5M).

### 3.5 The Downregulation of CTSL Expression Can Inhibit Tumour Growth

**FIGURE 6:**
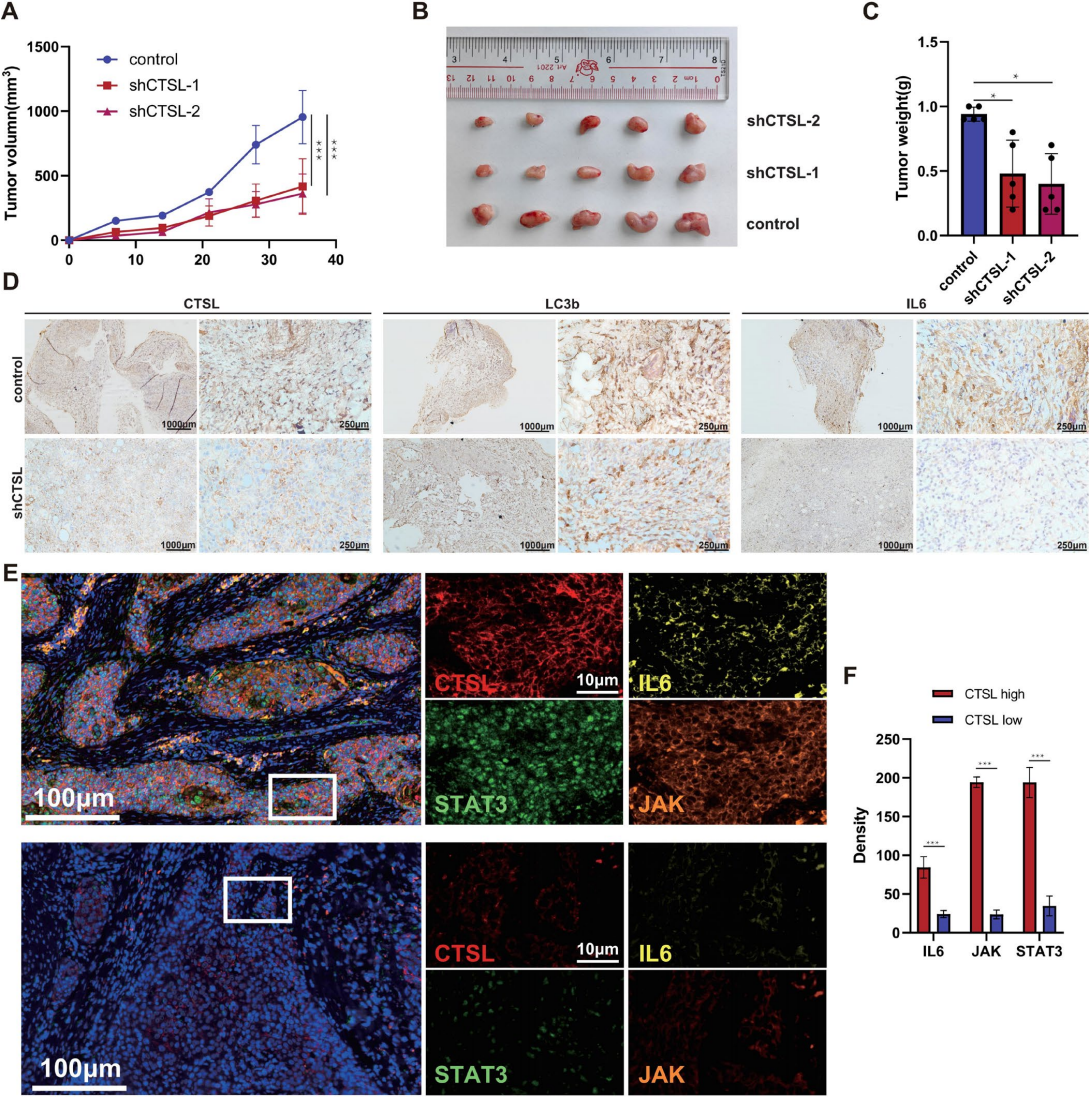
The downregulation of CTSL expression can inhibit tumour growth. (A) Tumour volume changes over time. Compared to the control group, the tumour volumes in the shCTSL‐1 and shCTSL‐2 groups show a significant decrease, and the difference is statistically significant. (B) Comparison of tumour volumes. (C) Comparison of tumour weights. (D) IHC detection of CTSL, LC3b and IL6 expression in the transplant tumours. The control group exhibits higher expression of CTSL, LC3b and IL6, while the shCTSL groups show lower expression. (E, F) Opal Multiplex Immuno‐Histochemistry and quantitative analysis of tissue sections from laryngeal cancer patients.

We subcutaneously injected stable CTSL‐knockdown TU212 laryngeal cancer cells (shCTSL‐1, shCTSL‐2) and control cells into nude mice and monitored tumour volumes every 7 days, as shown in Figure 6A–C. Compared to the control group, tumours induced by CTSL‐knockdown TU212 laryngeal cancer cells exhibited significant reduction in size, indicating that downregulation of CTSL can decrease tumour volume in vivo. Additionally, immunohistochemistry (IHC) confirmed that the levels of IL6 and LC3b in the CTSL‐knockdown group were significantly lower than those in the control group, further suggesting that CTSL promotes laryngeal cancer autophagy through the IL6‐JAK‐STAT3 signalling pathway (Figure 6D). Subsequently, we performed Opal Multiplex Immuno‐Histochemistry on tissue sections from laryngeal cancer patients and found a close relationship between CTSL and IL6, JAK and STAT3. This is consistent with previous studies (Figure 6E,F).

### 3.6 The Prognostic Value of CTSL‐Related Autophagy Genes in HNSC

**FIGURE 7:**
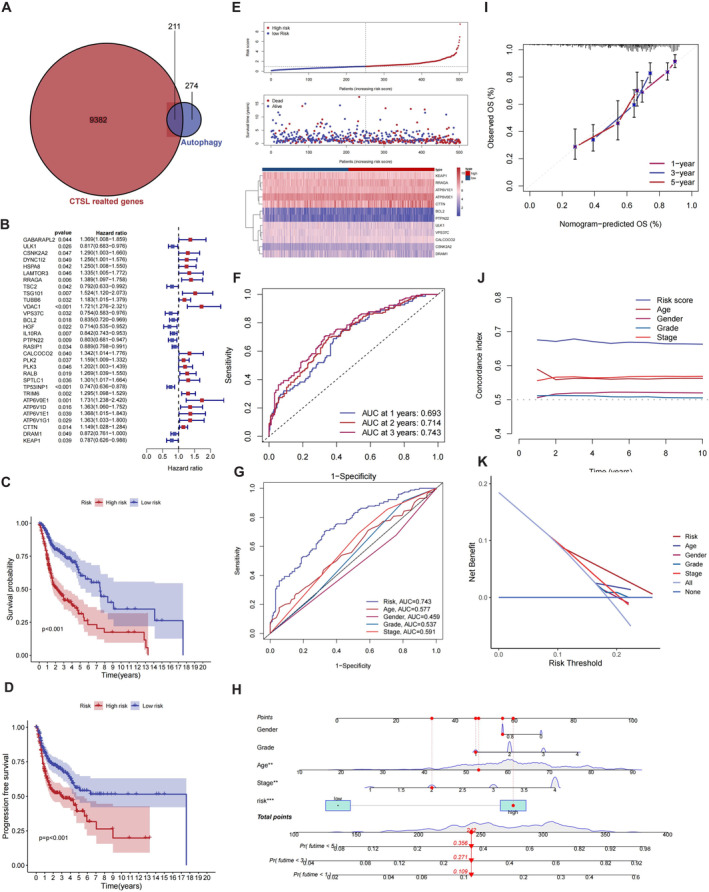
The prognostic value of CTSL‐related autophagy genes in HNSC. (A) Acquisition of 211 CTSL‐related autophagy genes. (B) Univariate Cox analysis identified 31 genes closely associated with survival. (C–E) Kaplan–Meier survival curves illustrate significant differences in overall survival (OS) and progression‐free survival (PFS) between high‐ and low‐risk groups based on the risk scores established by the CTSL‐related autophagy gene signature. Additionally, as the risk score increases, patient survival rates gradually decrease. (F, G) Receiver Operating Characteristic (ROC) curves for risk scores. (H) Successful establishment of a nomogram. (I–K) Calibration curves for risk scores, time‐dependent Concordance Index (C‐index) and Decision Curve Analysis (DCA) curves.

We aimed to enhance the prognostic accuracy of CTSL by considering its biological functions. Given the crucial role of CTSL in regulating autophagy, we initially identified 211 autophagy‐related genes closely associated with CTSL. Subsequently, through univariate Cox analysis, we narrowed down the pool to 31 survival genes (Figure 7A,B). A multifactorial Cox analysis was then performed on these 31 survival genes, yielding a signature composed of 12 autophagy‐related genes: ULK1, CSNK2A2, RRAGA, VPS37C, BCL2, PTPN22, CALCOCO2, ATP6V0E1, ATP6V1E1, CTTN, DRAM1, KEAP1. Next, we utilised a risk scoring algorithm, evaluating the overall survival risk by combining the expression levels of each included gene with its survival coefficient. The derived risk score held significant clinical relevance. Kaplan–Meier survival curves demonstrated a significant correlation between the CTSL‐associated autophagy gene signature and Overall Survival (OS) and Progression‐Free Survival (PFS) in HNSC. As the risk score increased, patient survival rates gradually decreased, establishing the signature as an independent predictor for HNSC prognosis (Figure 7C–E). The Area Under the Curve (AUC) of the ROC curve for the risk score was 0.693, 0.714 and 0.743 at 1, 3 and 5 years, respectively (Figure 7F). Compared to other clinically relevant features, the risk score exhibited the highest AUC value, demonstrating good calibration and clinical applicability (Figure 7G,I,K). Additionally, it displayed excellent discriminative ability across multiple time periods (Figure 7J). Combining the risk score with other clinical features, we established a nomogram to predict patient survival probabilities (Figure 7H).

### 3.7 The Value of the CTSL‐Related Autophagy Gene Signature

**FIGURE 8:**
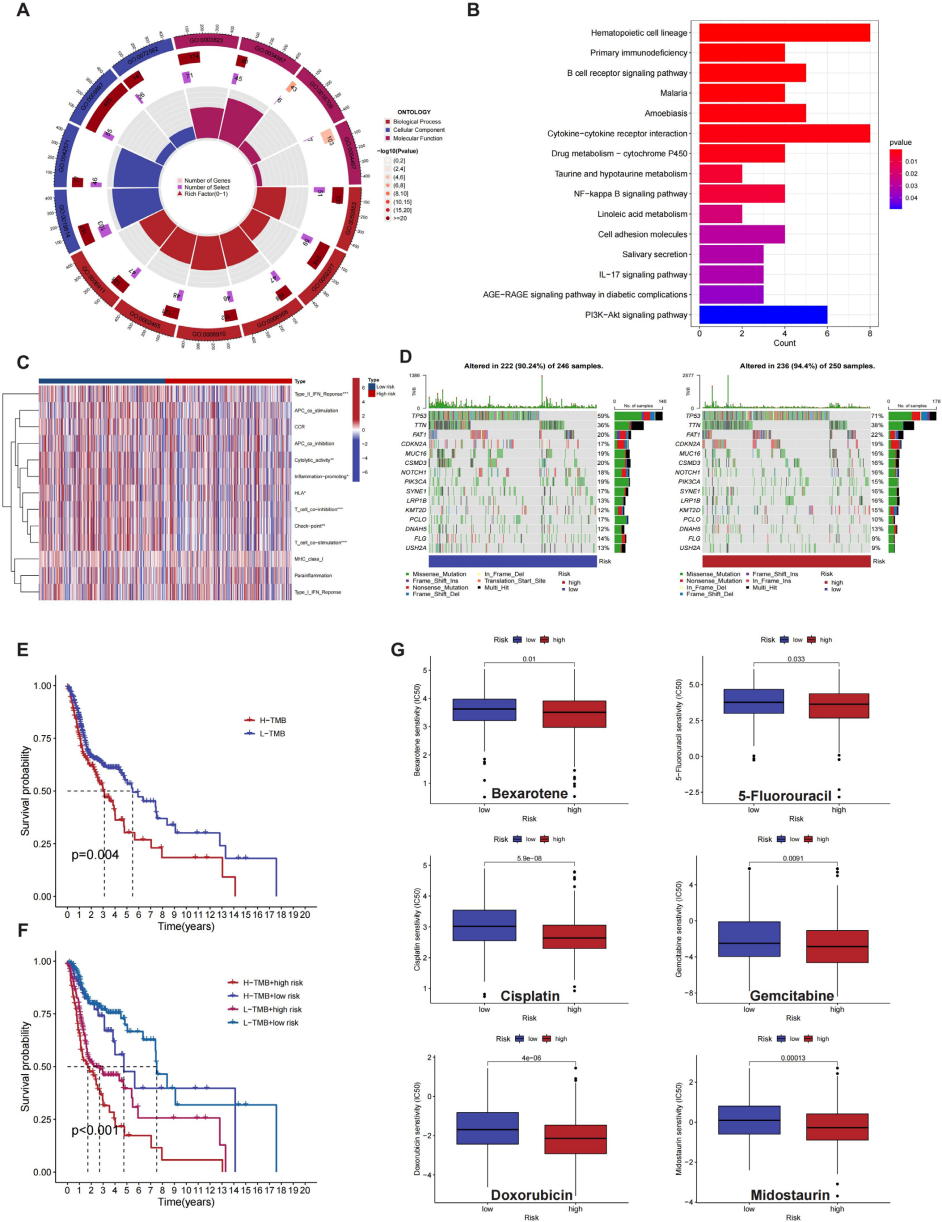
The value of the CTSL‐related Autophagy Gene Signature. (A, B) GO and KEGG enrichment analysis of differentially expressed genes between high‐ and low‐risk groups. (C) GSVA analysis of immune‐related gene sets. (D) Mutation frequency of high‐ and low‐risk groups. (E, F) Clinical significance of tumour mutation burden (TMB) combined with risk scores, as well as the tumour mutation load. (G) Drug sensitivity analysis.

To further explore the value of the CTSL related autophagy gene signature, we investigated the potential functions of differentially expressed genes in high‐ and low‐risk score groups through GO and Kyoto Encyclopaedia of Genes and Genomes (KEGG) analyses. In the GO analysis of BPs, the differentially expressed genes were primarily involved in B cell receptor signalling pathway, immunoglobulin production, complement activation (classical pathway), phagocytosis recognition, humoral immune response mediated by circulating immunoglobulin, and phagocytosis engulfment. The results of cellular component analysis showed associations with immunoglobulin complex, circulating immunoglobulin complex, external side of the plasma membrane, and blood microparticle. Molecular functional analysis indicated localization in antigen binding, immunoglobulin receptor binding, oxidoreductase activity and monooxygenase activity (Figure 8A). In the KEGG analysis, the differentially expressed genes were mainly associated with B cell receptor signalling pathway, Cytokine‐cytokine receptor interaction, IL‐17 signalling pathway and PI3K‐Akt signalling pathway, among others (Figure 8B). Gene Set Variation Analysis (GSVA) of immune‐related gene sets showed differences in immune functions between the high‐ and low‐risk groups, particularly in Type II IFN response, cytolytic activity, inflammation‐promoting genes, T cell co‐inhibition, T cell co‐stimulation and checkpoint genes (Figure 8C). Moreover, compared to the low‐risk group's mutation frequency of 90.24%, the high‐risk group exhibited a higher mutation frequency of 94.4% (Figure 8D). There was a significant survival difference between high‐ and low tumour mutation burden (TMB) groups, and the combination of risk score and TMB also demonstrated significant clinical significance (Figure 8E,F). We also examined whether the risk score could predict clinical drug responses. Bexarotene, Cisplatin, Doxorubicin, 5‐Fluorouracil, Gemcitabine and Midostaurin are commonly used cancer therapeutic drugs. Identifying patients suitable for these therapies has been a challenging task, as only a subset of patients truly benefits from conventional cancer treatments. To better predict treatment outcomes, reliable biomarkers are urgently needed. Fortunately, advancements in next‐generation sequencing and predictive methods have allowed us to predict clinical chemotherapy responses based on gene expression levels. IC50 values indicated a significantly greater response in the high‐risk group to Bexarotene, Cisplatin, Doxorubicin, 5‐Fluorouracil, Gemcitabine and Midostaurin (Figure 8G). These results suggest that this risk score has great potential for predicting prognosis and drug sensitivity.

## 4 Discussion

Autophagy is a primary mechanism that mediates the delivery of various cellular cargoes to lysosomes for degradation and recycling [19, 20]. In the initiation phase of autophagy, the autophagy complex containing beclin‐1 promotes the expansion of autophagosomes. Autophagy‐related genes, including ATG5, ATG7, ATG12, ATG16L1, among others, form complexes that bind to phosphatidylethanolamine, transforming into lipidated forms (LC3b), which are crucial for autophagosome formation [21, 22]. Additionally, p62, a multifunctional protein serving as an autophagic substrate, can be degraded through the autophagic pathway [23]. Laryngeal cancer, one of the deadliest malignancies worldwide, remains highly complex. Our understanding of laryngeal cancer is still incomplete, emphasising the need for in‐depth and sustained research to elucidate its potential mechanisms. Discovering new biomarkers and potential therapeutic targets is crucial for improving the prognosis of laryngeal cancer patients.

Cathepsin L has been extensively studied for its unique role, particularly in the occurrence of autophagy in various BPs. Research has confirmed that CTSL mediates autophagy in cervical cancer cells [15, 24], and Du et al.'s study suggests a close association between CTSL and chemoresistance in neuroblastoma [25]. Despite CTSL being reported to regulate BPs in many tumours, its role in laryngeal cancer remains unclear.

We systematically analysed the expression differences of CTSL in tumour and normal tissues using large databases and human tissue samples, confirming significantly higher CTSL expression in tumour tissues compared to normal tissues. Patients with elevated CTSL expression had worse prognoses. Furthermore, upregulation of CTSL promoted the proliferation and invasion of laryngeal cancer cells, while downregulation had inhibitory effects on tumour growth. CIBERSORTs and ESTIMATE algorithms revealed a close correlation between CTSL expression and immunity. In vitro experiments also confirmed that reduced CTSL expression inhibited tumour growth.

Autophagy is a highly conserved process implicated in the degradation of organelle fragments in lysosomes, considered a second type of programmed cell death [26]. Lysosomal proteases play a key role in the clearance process of autophagosomes. Autophagy degrades and recycles damaged proteins and organelles within the cell through the formation of autophagosomes and the fusion with lysosomes. Various proteases in lysosomes, such as cysteine proteases, aspartic proteases and serine proteases, work together in an acidic environment to effectively degrade substances within autophagosomes. The activity of lysosomal proteases is regulated by factors such as pH and phosphorylation, and their dysfunction is associated with various diseases, including neurodegenerative diseases and cancer [27, 28, 29, 30, 31]. Combining bioinformatics and cell experiments, we observed enhanced autophagy in laryngeal cancer tissues and cells. Upregulation of CTSL facilitated autophagy in laryngeal cancer cells, whereas downregulation had inhibitory effects. Co‐immunoprecipitation experiments also validated the interaction between CTSL and ATG7.

However, Lysosomal tissue proteases play important roles outside the cell, including promoting tissue remodelling and wound healing, participating in immune responses, regulating intercellular signalling, and influencing pathological conditions such as tumours and inflammation. They exhibit multiple biological functions through mechanisms such as degrading the extracellular matrix, participating in antigen presentation, regulating the release of cytokines and promoting cell invasion and metastasis in the tumour microenvironment. More related research needs to be conducted in the future [32].

Several signalling pathways have been reported to play crucial roles in regulating autophagy, including P53, PI3K, RAS and JAK–STAT pathways [6]. The IL6‐JAK‐STAT3 signalling pathway has a significant impact on various BPs in tumours, such as migration, invasion and angiogenesis [33, 34]. Targeting the IL6‐JAK‐STAT3 pathway is a promising therapeutic approach with multifaceted mechanisms [35, 36]. Through GSEA analysis, we revealed the mechanism by which CTSL acts through the IL6‐JAK‐STAT3 pathway in head and neck tumours. The activity of the IL6‐JAK‐STAT3 pathway was upregulated in laryngeal cancer tissues and cells. Knockdown of IL6 weakened the autophagy of CTSL‐overexpressing laryngeal cancer cells, while the addition of recombinant IL6 protein enhanced autophagy in CTSL‐overexpressing cells. In vitro experiments also confirmed the downregulation of IL6 and LC3b expression when CTSL expression was reduced. Interestingly, we found that CTSL promotes autophagy in laryngeal cancer through the IL6‐JAK‐STAT3 pathway, but IL6 does not affect CTSL expression. This may be because CTSL might regulate the expression of IL6 upstream, while IL6 is not an upstream regulator of CTSL. This suggests a unidirectional regulatory relationship. Additionally, the complex differences and regulations within the tumour microenvironment may also contribute to this phenomenon. The IL‐6 receptor (IL6R) plays a crucial role in various diseases, particularly in the tumour microenvironment and inflammatory responses. Studies indicate that IL‐6 activates the JAK‐STAT3 signalling pathway by binding to IL6R, thereby influencing the autophagic process in cells. The activation of this pathway not only promotes the survival and proliferation of tumour cells but may also regulate tumour resistance. In cancer prognosis, the expression level of IL6R is considered an important biomarker. High expression of IL6R is associated with tumour progression, metastasis and poor prognosis. Therefore, targeting IL6R may represent a new strategy for treating certain cancers [37, 38, 39]. In summary, we found that CTSL promotes autophagy in laryngeal cancer through the IL6‐JAK‐STAT3 signalling pathway.

Given the urgent need to discover new tumour prognostic biomarkers to guide effective personalised treatment interventions, we also assessed the prognostic value of CTSL‐related autophagy genes. Currently, single‐gene biomarkers often fail to capture the molecular heterogeneity of tumours and accurately predict cancer prognosis. This challenge has stimulated the development of various gene signatures [40]. Our study established an autophagy‐related signature consisting of 12 genes (ULK1, CSNK2A2, RRAGA, VPS37C, BCL2, PTPN22, CALCOCO2, ATP6V0E1, ATP6V1E1, CTTN, DRAM1, KEAP1) and validated the prognostic value of this signature in TCGA cohorts. The superiority of this risk score was confirmed through AUC curves, calibration curves, DCA curves and nomograms. Analysis of high‐ and low‐risk score groups revealed enrichment in multiple functional pathways, as well as differences in immune function and tumour mutation burden, indicating the guiding significance of the risk score in predicting the prognosis of laryngeal cancer patients. Moreover, the risk score was correlated with chemotherapy sensitivity. Therefore, CTSL may influence immune responses by regulating immune cell functions or altering the tumour microenvironment. This risk stratification could guide treatment strategies, and future studies could further explore the relationship between CTSL, this risk score and immune response and treatment outcomes in laryngeal cancer patients.

This study has certain limitations, such as not thoroughly investigating the specific reasons for the upregulation of CTSL in laryngeal cancer. Additionally, most findings come from western blot and qPCR analyses, and the sample size of laryngeal cancer patients is relatively small.

In conclusion, our study suggests that CTSL promotes autophagy in laryngeal cancer by regulating the IL6‐JAK‐STAT3 signalling pathway. We validated the association of CTSL with the prognosis and autophagy of laryngeal cancer across multiple cohorts. Furthermore, by considering the autophagy regulatory role of CTSL, we established an autophagy‐related signature, enhancing the ability of CTSL to predict the prognosis and drug sensitivity of laryngeal cancer. Although we have elucidated the mechanism by which CTSL promotes autophagy in laryngeal cancer cells by activating the IL6‐JAK‐STAT3 signalling pathway at the molecular and genetic levels, increasing evidence suggests that cancer represents a complex pathological ecosystem. In Weiren Luo's study [41], Nasopharyngeal carcinoma (NPC), a unique type of head and neck cancer, is proposed not as a hereditary disease but as an ecological one—a multidimensional, spatiotemporal pathological ecosystem exemplifying the ‘unity of ecology and evolution.’ NPC cells act as invasive species, and their metastasis represents multidirectional ecological dispersal. The progression of NPC can be explained using foundational ecological principles. The ‘Sangji Fish Pond’ model effectively illustrates the dynamic mutualistic relationships within the cancer ecosystem. Weiren Luo posits that ‘nothing in cancer evolution or ecology makes sense except in the light of the other.’ The cancer eco‐tree model he developed comprehensively points to future research directions. The establishment of the NPC ecological theory and cancer eco‐tree may provide a novel conceptual framework and paradigm for understanding cancer's complex pathogenic processes, as well as potential applications in prevention and treatment for patients. Therefore, in future research, it will be essential to continue elucidating the molecular mechanisms by which CTSL impacts patients with laryngeal cancer, while also focusing on studies of the complex pathological ecosystem associated with laryngeal cancer, with the aim of providing support and improved outcomes for patients with laryngeal cancer.

## Author Contributions

**Xueying Wang:** data curation (equal), methodology (equal), supervision (equal), writing – original draft (equal). **Junrong Wang:** writing – original draft (equal), writing – review and editing (equal). **Lei Wang:** writing – original draft (equal), writing – review and editing (equal). **Ming Song:** writing – original draft (equal), writing – review and editing (equal). **Hongxue Meng:** writing – review and editing (equal). **Erliang Guo:** writing – review and editing (equal). **Susheng Miao:** data curation (equal), project administration (equal), resources (equal), supervision (equal), writing – review and editing (equal).

## Ethics Statement

This study was approved by the Medical Ethics Review Committee of Harbin Medical University. All procedures in this study were conducted in accordance with the ethics committee approved protocols.

## Consent

Written informed consent was obtained from the patients for their anonymized information to be published in this article.

## Conflicts of Interest

The authors declare no conflicts of interest.

## Supporting information


Appendix S1.

## Data Availability

The data that support the findings of this study are available in GEO at https://www.ncbi.nlm.nih.gov/geo/, reference number GSE58911, GSE58319, GSE84957, GSE41613, GSE65858.

## References

[1] R. L. Siegel , K. D. Miller , H. E. Fuchs , et al., “Cancer Statistics, 2022,” CA: A Cancer Journal for Clinicians 72, no. 1 (2022): 7–33. 35020204 10.3322/caac.21708

[2] N. KarichE , M. T. Hortal , S. Benyahia , et al., “Comparative Assessment of HPV, Alcohol and Tobacco Etiological Fractions in Algerian Patients With Laryngeal Squamous Cell Carcinoma,” Infect Agent Cancer 13 (2018): 8. 29563964 10.1186/s13027-018-0181-x PMC5851087

[3] N. Vigneswaran and M. D. Williams , “Epidemiologic Trends in Head and Neck Cancer and Aids in Diagnosis,” Oral and Maxillofacial Surgery Clinics of North America 26, no. 2 (2014): 123–141. 24794262 10.1016/j.coms.2014.01.001 PMC4040236

[4] C. R. Leemans , P. J. F. Snijders , and R. H. Brakenhoff , “The Molecular Landscape of Head and Neck Cancer,” Nature Reviews. Cancer 18, no. 5 (2018): 269–282. 29497144 10.1038/nrc.2018.11

[5] C. E. Steuer , M. El‐Deiry , J. R. Parks , et al., “An Update on Larynx Cancer,” CA: a Cancer Journal for Clinicians 67, no. 1 (2017): 31–50. 27898173 10.3322/caac.21386

[6] J. M. M. Levy , C. G. Towers , and A. Thorburn , “Targeting Autophagy in Cancer,” Nature Reviews. Cancer 17, no. 9 (2017): 528–542. 28751651 10.1038/nrc.2017.53 PMC5975367

[7] J. Debnath , N. Gammoh , and K. M. Ryan , “Autophagy and Autophagy‐Related Pathways in Cancer,” Nature Reviews. Molecular Cell Biology 24, no. 8 (2023): 560–575. 36864290 10.1038/s41580-023-00585-z PMC9980873

[8] M. Marinkovic , M. Sprung , M. Buljubasic , et al., “Autophagy Modulation in Cancer: Current Knowledge on Action and Therapy,” Oxidative Medicine and Cellular Longevity 2018 (2018): 8023821. 29643976 10.1155/2018/8023821 PMC5831833

[9] M. L. Han , Y. F. Zhao , C. H. Tan , et al., “Cathepsin L Upregulation‐Induced EMT Phenotype Is Associated With the Acquisition of Cisplatin or Paclitaxel Resistance in A549 Cells,” Acta Pharmacologica Sinica 37, no. 12 (2016): 1606–1622. 27840408 10.1038/aps.2016.93 PMC5309731

[10] Q. Zhang , M. Han , W. Wang , et al., “Downregulation of Cathepsin L Suppresses Cancer Invasion and Migration by Inhibiting Transforming Growth Factor‐Beta‐Mediated Epithelial‐Mesenchymal Transition,” Oncology Reports 33, no. 4 (2015): 1851–1859. 25632968 10.3892/or.2015.3754

[11] C. Tyagi , S. Grover , J. Dhanjal , et al., “Mechanistic Insights Into Mode of Action of Novel Natural Cathepsin L Inhibitors,” BMC Genomics 14 Suppl 8, no. Suppl 8 (2013): S10. 10.1186/1471-2164-14-S8-S10 PMC4042235 24564425

[12] K. Miyamoto , M. Iwadate , Y. Yanagisawa , et al., “Cathepsin L Is Highly Expressed in Gastrointestinal Stromal Tumors,” International Journal of Oncology 39, no. 5 (2011): 1109–1115. 21769426 10.3892/ijo.2011.1127

[13] F. C. Sigloch , M. Tholen , A. Gomez‐Auli , et al., “Proteomic Analysis of Lung Metastases in a Murine Breast Cancer Model Reveals Divergent Influence of CTSB and CTSL Overexpression,” Journal of Cancer 8, no. 19 (2017): 4065–4074. 29187882 10.7150/jca.21401 PMC5706009

[14] X. Zheng , P. M. Chou , B. L. Mirkin , et al., “Senescence‐Initiated Reversal of Drug Resistance: Specific Role of Cathepsin L,” Cancer Research 64, no. 5 (2004): 1773–1780. 14996739 10.1158/0008-5472.can-03-0820

[15] X. Zheng , F. Chu , P. M. Chou , et al., “Cathepsin L Inhibition Suppresses Drug Resistance In Vitro and In Vivo: A Putative Mechanism,” American Journal of Physiology. Cell Physiology 296, no. 1 (2009): C65–C74. 18971393 10.1152/ajpcell.00082.2008 PMC4042848

[16] H. L. Olsvik , S. Svenning , Y. P. Abudu , et al., “Endosomal Microautophagy Is an Integrated Part of the Autophagic Response to Amino Acid Starvation,” Autophagy 15, no. 1 (2019): 182–183. 30295124 10.1080/15548627.2018.1532265 PMC6287684

[17] N. M. Mazure and J. Pouyssegur , “Hypoxia‐Induced Autophagy: Cell Death or Cell Survival?,” Current Opinion in Cell Biology 22, no. 2 (2010): 177–180. 20022734 10.1016/j.ceb.2009.11.015

[18] B. Levine and G. Kroemer , “Autophagy in the Pathogenesis of Disease,” Cell 132, no. 1 (2008): 27–42. 18191218 10.1016/j.cell.2007.12.018 PMC2696814

[19] D. J. Klionsky , G. Petroni , R. K. Amaravadi , et al., “Autophagy in Major Human Diseases,” EMBO Journal 40, no. 19 (2021): e108863. 34459017 10.15252/embj.2021108863 PMC8488577

[20] A. J. Clarke and A. K. Simon , “Autophagy in the Renewal, Differentiation and Homeostasis of Immune Cells,” Nature Reviews. Immunology 19, no. 3 (2019): 170–183. 10.1038/s41577-018-0095-2 30531943

[21] Y. G. Zhao , P. Codogno , and H. Zhang , “Machinery, Regulation and Pathophysiological Implications of Autophagosome Maturation,” Nature Reviews. Molecular Cell Biology 22, no. 11 (2021): 733–750. 34302147 10.1038/s41580-021-00392-4 PMC8300085

[22] H. Nakatogawa , “Mechanisms Governing Autophagosome Biogenesis,” Nature Reviews. Molecular Cell Biology 21, no. 8 (2020): 439–458. 32372019 10.1038/s41580-020-0241-0

[23] H. Wei , C. Wang , C. M. Croce , and J. L. Guan , “p62/SQSTM1 Synergizes With Autophagy for Tumor Growth In Vivo,” Genes & Development 28, no. 11 (2014): 1204–1216. 24888590 10.1101/gad.237354.113 PMC4052766

[24] L. Galluzzi and D. R. Green , “Autophagy‐Independent Functions of the Autophagy Machinery,” Cell 177, no. 7 (2019): 1682–1699. 31199916 10.1016/j.cell.2019.05.026 PMC7173070

[25] X. Du , L. Ding , S. Huang , et al., “Cathepsin L Promotes Chemresistance to Neuroblastoma by Modulating Serglycin,” Frontiers in Pharmacology 13 (2022): 920022. 36133820 10.3389/fphar.2022.920022 PMC9484481

[26] G. J. Yoshida , “Therapeutic Strategies of Drug Repositioning Targeting Autophagy to Induce Cancer Cell Death: From Pathophysiology to Treatment,” Journal of Hematology & Oncology 10, no. 1 (2017): 67. 28279189 10.1186/s13045-017-0436-9 PMC5345270

[27] N. Mizushima and M. Komatsu , “Autophagy: Renovation of Cells and Tissues,” Cell 147, no. 4 (2011): 728–741. 22078875 10.1016/j.cell.2011.10.026

[28] Z. Yang and D. J. Klionsky , “Eaten Alive: A History of Macroautophagy,” Nature Cell Biology 12, no. 9 (2010): 814–822. 20811353 10.1038/ncb0910-814 PMC3616322

[29] G. Kroemer and M. Jaattela , “Lysosomes and Autophagy in Cell Death Control,” Nature Reviews. Cancer 5, no. 11 (2005): 886–897. 16239905 10.1038/nrc1738

[30] C. Settembre , A. Fraldi , D. L. Medina , et al., “Signals From the Lysosome: A Control Centre for Cellular Clearance and Energy Metabolism,” Nature Reviews. Molecular Cell Biology 14, no. 5 (2013): 283–296. 23609508 10.1038/nrm3565 PMC4387238

[31] S. R. Yoshii and N. Mizushima , “Monitoring and Measuring Autophagy,” International Journal of Molecular Sciences 18, no. 9 (2017): 1865. 28846632 10.3390/ijms18091865 PMC5618514

[32] O. C. Olson and J. A. Joyce , “Cysteine Cathepsin Proteases: Regulators of Cancer Progression and Therapeutic Response,” Nature Reviews. Cancer 15, no. 12 (2015): 712–729. 26597527 10.1038/nrc4027

[33] R. Siersbaek , V. Scabia , S. Nagarajan , et al., “IL6/STAT3 Signaling Hijacks Estrogen Receptor Alpha Enhancers to Drive Breast Cancer Metastasis,” Cancer Cell 38, no. 3 (2020): 412–423.e9. 32679107 10.1016/j.ccell.2020.06.007 PMC7116707

[34] M. S. Pan , H. Wang , K. H. Ansari , et al., “Gallbladder Cancer‐Associated Fibroblasts Promote Vasculogenic Mimicry Formation and Tumor Growth in Gallbladder Cancer via Upregulating the Expression of NOX4, a Poor Prognosis Factor, Through IL‐6‐JAK‐STAT3 Signal Pathway [J],” Journal of Experimental & Clinical Cancer Research 39, no. 1 (2020): 234. 33153467 10.1186/s13046-020-01742-4 PMC7643415

[35] D. Y. Oh , S. H. Lee , S. W. Han , et al., “Phase I Study of OPB‐31121, an Oral STAT3 Inhibitor, in Patients With Advanced Solid Tumors,” Cancer Research and Treatment 47, no. 4 (2015): 607–615. 25715763 10.4143/crt.2014.249 PMC4614199

[36] X. Liu , G. W. Jones , E. H. Choy , et al., “The Biology Behind Interleukin‐6 Targeted Interventions,” Current Opinion in Rheumatology 28, no. 2 (2016): 152–160. 26751841 10.1097/BOR.0000000000000255

[37] M. Y. Taher , D. M. Davies , and J. Maher , “The Role of the Interleukin (IL)‐6/IL‐6 Receptor Axis in Cancer,” Biochemical Society Transactions 46, no. 6 (2018): 1449–1462. 30467123 10.1042/BST20180136

[38] L. Sreenivasan , H. Wang , S. Q. Yap , et al., “Autocrine IL‐6/STAT3 Signaling Aids Development of Acquired Drug Resistance in Group 3 Medulloblastoma,” Cell Death & Disease 11, no. 12 (2020): 1035. 33279931 10.1038/s41419-020-03241-y PMC7719195

[39] N. Yanaihara , Y. Hirata , N. Yamaguchi , et al., “Antitumor Effects of Interleukin‐6 (IL‐6)/interleukin‐6 Receptor (IL‐6R) Signaling Pathway Inhibition in Clear Cell Carcinoma of the Ovary,” Molecular Carcinogenesis 55, no. 5 (2016): 832–841. 25856562 10.1002/mc.22325

[40] P. Ahluwalia , R. Kolhe , and G. K. Gahlay , “The Clinical Relevance of Gene Expression Based Prognostic Signatures in Colorectal Cancer,” Biochimica Et Biophysica Acta. Reviews on Cancer 1875, no. 2 (2021): 188513. 33493614 10.1016/j.bbcan.2021.188513

[41] W. Luo , “Nasopharyngeal Carcinoma Ecology Theory: Cancer as Multidimensional Spatiotemporal “Unity of Ecology and Evolution” Pathological Ecosystem,” Theranostics 13, no. 5 (2023): 1607–1631. 37056571 10.7150/thno.82690 PMC10086202

