# Supplementary material for: CTSL Promotes Autophagy in Laryngeal Cancer Through the IL6‐JAK‐STAT3 Signalling Pathway
Source: J Cell Mol Med. 2025 Feb 2;29(3):e70364. doi: 10.1111/jcmm.70364 (PMC11787816; doi:10.1111/jcmm.70364)
Supplement: Supplementary file 1 — Appendix S1. [file JCMM-29-e70364-s001.docx]

tumor mutation load. G: Drug sensitivity analysis.

|  | Forward Primer | Reverse Primer |
| --- | --- | --- |
| CTSL | CACCGGCTTTGTGGACATC | ATGACCTGCATCAATAGCAACA |
| ATG7 | ATGATCCCTGTAACTTAGCCCA | CACGGAAGCAAACAACTTCAAC |
| ATG5 | AGAAGCTGTTTCGTCCTGTGG | AGGTGTTTCCAACATTGGCTC |
| SQSTM1 | GACTACGACTTGTGTAGCGTC | AGTGTCCGTGTTTCACCTTCC |
| ATG16L | TCTGGGACATTCGATCAGAGAG | CCTTTCTGGGTTTAAGTCCAGG |
| IL6 | CCTGAACCTTCCAAAGATGGC | TTCACCAGGCAAGTCTCCTCA |
| LC3B | AAGGCGCTTACAGCTCAATG | CTGGGAGGCATAGACCATGT |
| BECLIN1 | GGTGTCTCTCGCAGATTCATC | TCAGTCTTCGGCTGAGGTTCT |
| ATG12 | CTGCTGGCGACACCAAGAAA | CGTGTTCGCTCTACTGCCC |
| JAK | AGCCTATCGGCATGGAATATCT | TAACACTGCCATCCCAAGACA |
| STAT3 | ATCACGCCTTCTACAGACTGC | CATCCTGGAGATTCTCTACCACT |

**Table S1** The qRT-PCR sequenses


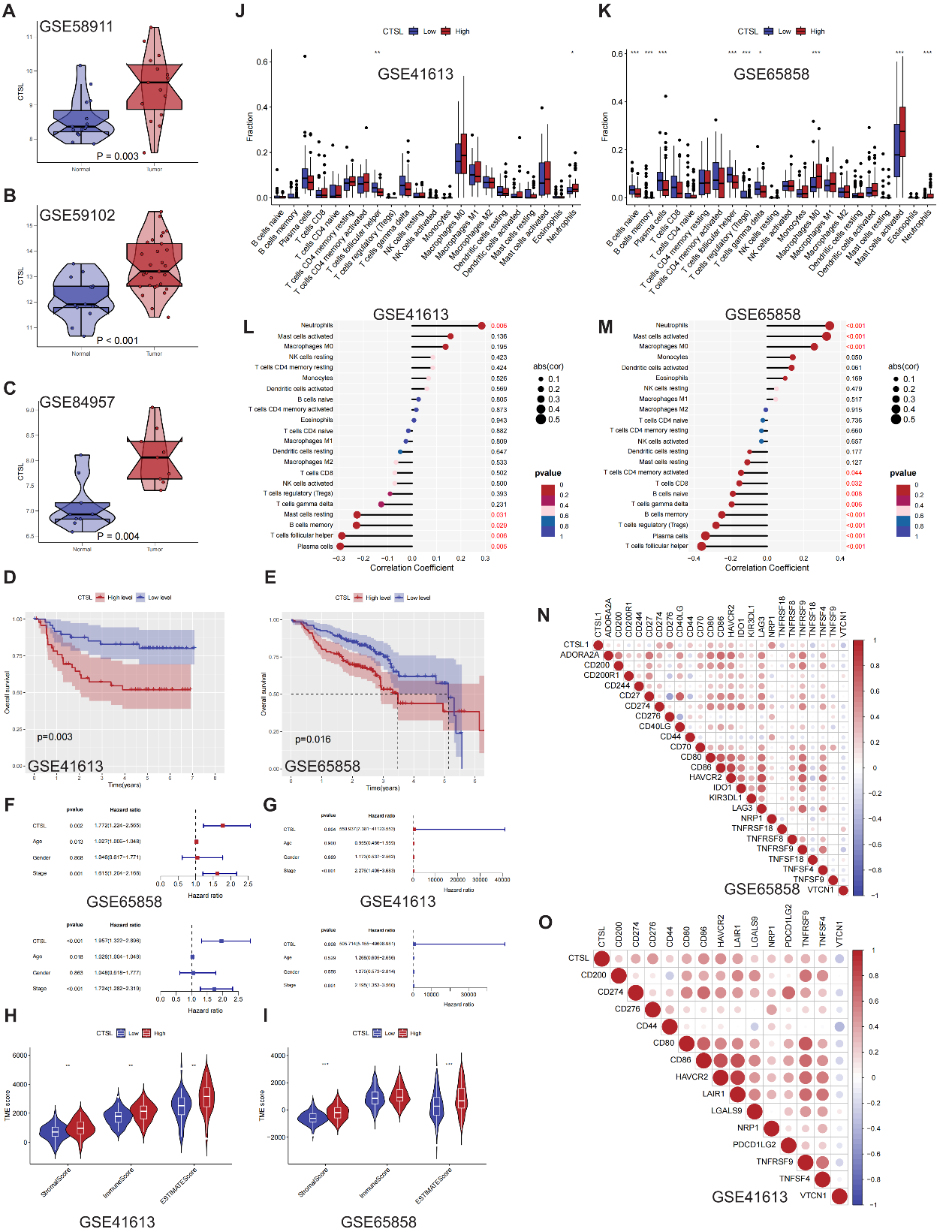


**Fig S1 Analyzing the correlation of CTSL with clinical and immune-related factors in GEO datasets.** A-C: Differential analysis of CTSL expression in cancer and adjacent tissues in GEO datasets. D, E: Kaplan-Meier survival curves for CTSL in GEO datasets showing its association with the survival of HNSC patients. F, G: Univariate analysis of CTSL in GEO datasets. H, I: Correlation analysis between CTSL and TME scores in GEO datasets. J-M: Correlation analysis of CTSL with immune cell infiltration in GEO datasets. N, O: Analysis of the correlation between CTSL and immune checkpoint expression in GEO datasets.
